# Content of phenolic compounds and vitamin C and antioxidant activity in wasted parts of Sudanese citrus fruits

**DOI:** 10.1002/fsn3.660

**Published:** 2018-05-08

**Authors:** Khitma A. Sir Elkhatim, Randa A. A. Elagib, Amro B. Hassan

**Affiliations:** ^1^ Environment and Natural Resource and Desertification Research Institute (ENDRI) National Center for Research Khartoum Sudan; ^2^ Present address: Faculty of Science Institute of Geography University of Bern Bern Switzerland

**Keywords:** antioxidants, citrus, flavonoids, peels, phenolic, pulp, vitamin C

## Abstract

Phenolic compounds, vitamin C, and the antioxidant activity of wasted parts of citrus (orange, lemon, and grapefruit) fruits were investigated. Ethanolic extracts from whole fruit, peel, and pulp containing seeds of each type of citruses were prepared. Within each type of citrus, results revealed that peels contained a higher amount of phenolic compound, flavonoids, vitamin C, and antioxidant activity than those of their inner wasted parts (pulp and seeds). Peels of grapefruit had the highest total phenolic content followed by lemon and orange, which was found to be 77.3, 49.8, and 35.6 mg of gallic acid equivalent/g of peels, respectively. In contrast, orange peels contain the highest amount of flavonoids (83.3 mg of catechin equivalent/g) and vitamin C (110.4 mg/100 g) compared to the peels of the other citrus fruit used in this study. In general, the high content of antioxidant capacity and activity of citrus waste, particularly the peels, indicated that they may impart health and nutritional benefit when involving in the food industry as a natural antioxidant.

## 1 INTRODUCTION

Recently, with the increment of food consumption as well as growing up of the food industry, the amount of food wastes, particularly wastes from fruit and vegetables, are increased. These wastes are considered as one of the main sources of municipal solid wastes (MSWs). Deng et al. (2012) stated that the accumulation of fruit and vegetable wastes such as citrus fruit peels, particularly in urban areas, causes a severe environmental problem. Consequently, upgrading systems for food waste reduction, specifically recycling, have been practiced for production of many useful products, such as utilization of fruit and vegetable wastes as a source of bioactive compounds. These compounds have high‐value products, and their recovery may be economically attractive (Ajila, Naidu, Bhat, & Prasada Rao, 2007; Hernandez‐Carranze et al., 2016; Kunradi et al., 2009). Moreover, these bioactive compounds, including phenolic compounds and other phytochemical compounds, have nutritional and health benefits for the humans. Besides health and nutritional benefits, antioxidants have important contributors to the food industry. They are capable to prevent the propagation reaction during the oxidation process, which results in maintaining the quality and shelf life of food products during handling and storage (Masuda, Inaba, & Takeda, 2001; Saito, Okamoto, & Kawabata, 2004).

In general, citrus fruits are considered as one of the natural resources of antioxidants, which contain an appreciable amount of ascorbic acid, flavonoids, and phenolic compounds (Al‐Juhaimi & Ghafoor, 2013; Ebrahimzadeh, Hosseinimehr, & Gayekhloo, 2004; Fernandez‐Lopez, Zhi, Aleson‐Carbonell, Perez‐ Alvarez, & Kuri, 2005; Jayaprakasha & Patil, 2007).

In food manufacturing, citrus is mainly used for producing fresh juice or citrus‐based drinks, so large amount of citrus waste such as peels, pulp, and seeds are formed annually. However, the enormous quantity of the bioactive compounds may present in the pulp, seeds, and peels of many vegetables and fruits (Vattem & Shetty, 2002). Hence, in the last decade, several studies suggested that citrus waste could be used as natural sources of antioxidants to take advantage of these wastes (Bocco, Cuvelier, Richard, & Berset, 1998; Llorach, Espin, Tomas‐Barberan, & Ferreres, 2003; Manthey & Grohmann, 2001; Wolfe, Wu, & Liu, 2003). Hence, estimation of the antioxidant power of the citrus fruit wasted part is required, so as to explore the potentials of their use in food manufacturing. Therefore, the aim of this study was to evaluate the capacity of phenolic, flavonoids compound, vitamin C as well as the antioxidant activity in wasted parts of Sudanese citrus cultivars (orange, lemon, and grapefruits) fruits and then compared to those of whole fruits.

## 2 MATERIALS AND METHODS

### 2.1 Collection and preparation of fruit sample

Three different citrus fruits (lemon, orange, and grapefruit) were obtained from the local market, Khartoum. The peels were removed manually, and then, the fruits were squeezed to separate juice from pulp and seeds. The separated parts were collected and dried using a freeze drier apparatus. The freeze‐dried samples were then grounded and kept at 4°C during the analysis.

### 2.2 Preparation of extracts

To prepare the extract, the milled sample was mixed with ethanol at a ratio of 1:25 (w/v) at ambient temperature and left to stand for 24 hr. The mixture was then filtered using a filter paper (Whatman No. 1). The residue was washed with ethanol, and the collected extract was dried under vacuum using a rotary evaporator and kept dry for further analysis. The extracts were reconstituted by pure ethanol directly before the analysis of total phenolic content and total flavonoids.

### 2.3 Determination of total phenolic content

The total phenolic content of the samples was determined by the Folin–Ciocalteu's reagent method (Waterhouse, 2001) with slight modifications. An aliquot (20 μl) of a dried extract ethanolic solution (1:10 w/v) was added to 1.58 ml water and 100 μl of the Folin–Ciocalteu reagent. After 5 min, 300 μl of the sodium carbonate solution was added to the mixture and carefully agitated for 10 min. The mixture was allowed to stand in the dark for 2 hr at 20°C. The absorption was measured at 765 nm using UV spectrophotometer. Different concentrations of gallic acid dissolved in pure ethanol were used to prepare the calibration curve (*R* ^2^ = .9672). The total phenolic content was expressed as milligrams of gallic acid equivalent per gram of dried peel, pulp, or whole fruit from individual citrus fruits (mg GAE/g DW).

### 2.4 Determination of total flavonoid content

Total flavonoid content (TFC) of the extracts was measured according to the colorimetric assay of Kim, Jeong, and Lee (2003). One milliliter of the ethanolic extract (1:10 w/v) was added to 300 μl sodium nitrite solution (5%) followed by 300 μl aluminum chloride (10%). The mixtures were incubated at room temperature for 5 min, and then, 2 ml of 1 mol/L sodium hydroxide was added. Immediately, the volume of reaction mixture was made to 10 ml with distilled water and then thoroughly vortexed. The absorbance of the mixture was determined at 510 nm. A calibration curve was prepared from different concentrations of catechin (*R* ^2^ = .974). Total flavonoid content was reported as milligrams of catechin equivalents per g dry weight sample (mg CE/g DW).

### 2.5 Determination of vitamin C

Vitamin C in the sample was determined by titrating its aqueous extract with a solution of 2,6‐dichlorophenol‐indophenol dye to a faint pink endpoint (AOAC, 2005).

### 2.6 Diphenyl‐2‐picrylhydrazyl (DPPH) scavenging assay

Scavenging activity of DPPH radicals of plant extracts was measured according to the method reported by Chang et al. (2001) cited from Shyura, Tsung, Chenb, Chiua, and Lo (2005) with minor modifications. Assays were performed in 3 ml reaction mixtures containing 2.0 ml of 0.1 mM DPPH‐ethanol solution, 0.9 ml of 50 mM Tris‐HCl buffer (pH 7.4), and 0.1 ml of deionized H_2_O (as control) or test plant extracts. After 30 min of incubation at room temperature, the absorbance of the reaction mixtures at 517 nm was taken. The inhibitory effect of DPPH was calculated according to the following formula:

$$\text{Inhibition}\;\left(\%\right)=\left[\frac{\text{Absorbance control}-\text{Absorbance sample}}{\text{Absorbance control}}\right]\times 100$$

### 2.7 Statistical analysis

All data were expressed as mean and standard deviation of three replicates. The data were conducted using a completely randomized block design and analyzed using two‐way analysis of variance (ANOVA). Significant differences were calculated at *p* < .05 using least significant difference (LSD).

## 3 RESULTS

### 3.1 Phenolic composition of citrus fruits

**Figure 1:**
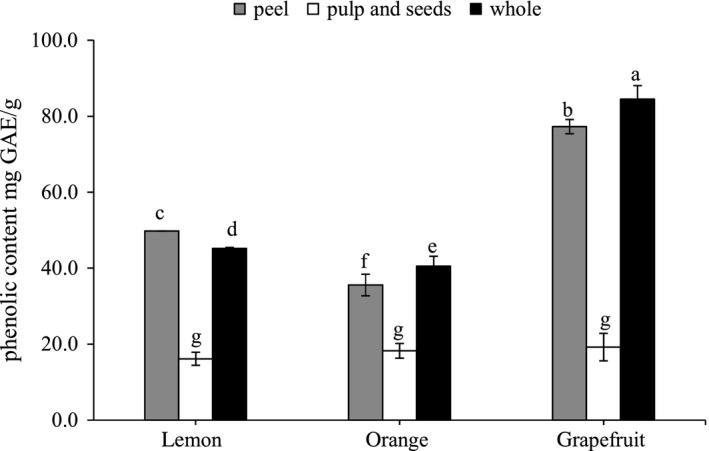
Phenolic content of wasted part extracts of lemon, orange, and grapefruit. Data represent the mean ± *SD* (*n* = 3). Values followed by the same letter are not significantly different (*p* < .05) as assessed by least significant difference (LSD)

The total phenolic contents of the peel, pulp containing seeds, and the whole fruit of lemon, orange, and grapefruits were expressed as mg of gallic acid equivalent per g dry weight (Figure 1). Among the citrus fruits, it was clearly observed that grapefruit significantly (*p* < .05) contained higher amount of phenolic compounds followed by lemon and orange fruits. The total phenolic content of the peels was found significantly (*p* < .05) higher in the grapefruit followed by lemon and orange. It was found to be 77.3, 49.8, and 35.6 mg of gallic acid equivalent/g of grapefruit, lemon, and orange peels, respectively. However, the total phenolic content of the inner wasted parts (pulp and seeds) was not significantly different (*p* < .05) among citrus fruit types. It ranged between 19.2 and 16.2 mg of gallic acid equivalent/g.

Obtained results show that the peels of lemon, orange, and grapefruit contain more phenolic compound compare to the pulp and seeds. Additionally, the phenolic content of grapefruit and its wasted parts was higher than those of orange and lemon. Moreover, our results were in agreement with those reported by several studies. Abeysinghe et al. (2007) and Goulas and Manganaris (2012) stated that peel of citrus fruit contains more bioactive compound than pulp. Furthermore, Li, Smith, and Hossain (2006) reported that peels of grapefruit contain higher total phenolic content than mandarin, yeb Ben lemon, orange, and meyer lemon peel.

**Figure 2:**
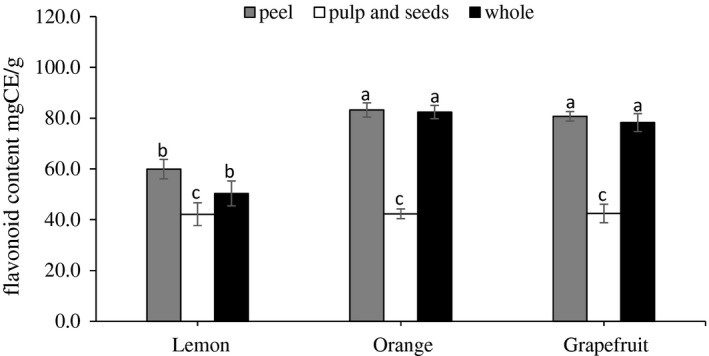
Flavonoid content of wasted part extracts of lemon, orange, and grapefruit. Data represent the mean ± *SD* (*n* = 3). Values followed by the same letter are not significantly different (*p* < .05) as assessed by least significant difference (LSD)

Figure 2 describes the total flavonoid content in the whole and wasted parts of citrus fruits. The obtained results indicated that both of grapefruit and orange fruits contained higher amount of the total flavonoids than those of lemon fruits. Additionally, the total flavonoid contents of orange and grapefruit peels were significantly (*p* < .05) higher than those of lemon's peel. The values of the total flavonoids in fruit peels were found to be 80.8, 83.3, and 59.9 mg of catechin equivalent/g for grapefruit, orange, and lemon, respectively. For the inner waste part (pulp and seeds), similar to the total phenolic content, the total flavonoids were not significantly different (*p* < .05) among the citrus fruit types.

Generally, within all citrus types, the peels tended to contain the greatest flavonoid content than in the pulp and seeds. In contrast to obtained results, Singh and Immanuel (2014) stated that total flavonoid content was found higher in lemon peel comparing to those of other citrus species such as orange and pomegranate. Such variation might be due to differences in the nature and origin of species and extraction solvent.

### 3.2 Vitamin C content of citrus fruits

**Figure 3:**
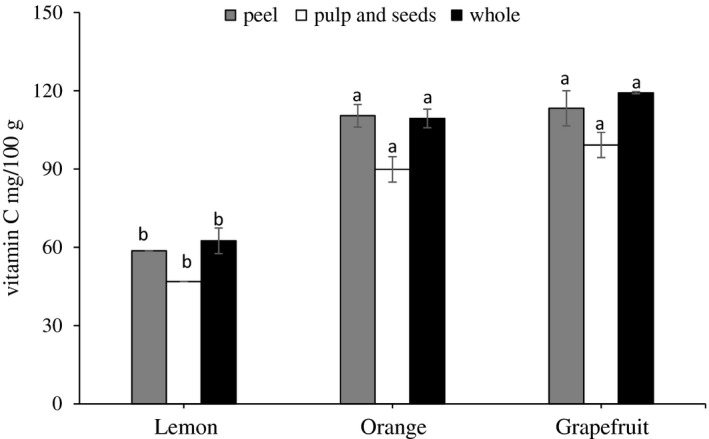
Vitamin C content of wasted part extracts of lemon, orange, and grapefruit. Data represent the mean ± *SD* (*n* = 3). Values followed by the same letter are not significantly different (*p* < .05) as assessed by least significant difference (LSD)

The vitamin C content in the lemon, orange, and grapefruit was quantified (Figure 3). From the figure, it was observed that the vitamin C content was found much higher in grapefruit followed by orange and lemon. Similar observation was reported that orange and grapefruit have the highest vitamin C content than lemon (Fatin Najwa & Azrina, 2017).

However, for each type of citrus fruit, no significant (*p* < .05) difference in vitamin C was observed among the fruit part.

Vitamin C content of grapefruit, orange, and lemon peels was found to be 113.3, 110.4, and 58.59 mg/100 g, respectively. While it was found to be 99.2, 89.8, and 46.9 mg/g in the inner parts (pulp and seeds) of grapefruit, orange, and lemon, respectively. In contrast to the obtained results, Barros, Ferreira, and Genovese (2012) reported that the pulp of different commercial citrus fruit from Brazil contained higher amount of ascorbic acid than in the peels. This variation in results might be due to the variation in the cultivars, maturity stages, and other environmental factors.

### 3.3 DPPH scavenging assay of citrus fruits

**Figure 4:**
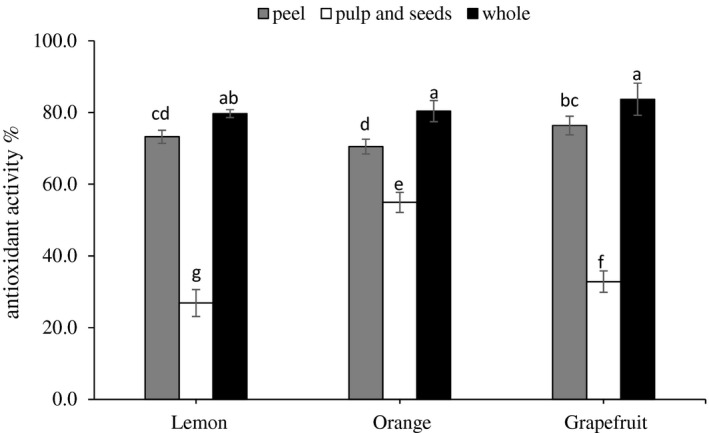
Antioxidant activity of wasted part extracts of lemon, orange, and grapefruit. Data represent the mean ± *SD* (*n* = 3). Values followed by the same letter are not significantly different (*p* < .05) as assessed by least significant difference (LSD)

The scavenging model of DPPH radical is widely used as a method for assessing antioxidant activity in a period relatively short compared to other methods (Wang, Jónsdóttir, & Ólafsdóttir, 2009). Figure 4 displayed the antioxidant activity of wasted part extracts of citrus fruits. In all types of fruits, the antioxidant activity is significantly (*p* < .05) higher in the peels than in the inner parts. For the peels, it was found significantly higher in grapefruit and lemon (76.4% and 73.2%, respectively) than in orange (70.5%). However, the antioxidant activity of orange's pulp and seeds was much higher than that of grapefruit and lemon.

The antioxidant activity in the peel of the three types of citrus fruits significantly (*p* < .05) higher than that in pulp and seeds. The high antioxidant activity in citrus peels could be related to the high total phenolic and flavonoid contents. Similar results were found in a previous publication (Molina‐Quijada, Medina‐Ju′arez, Gonz′alez‐Aguilar, Robles‐S′anchez, & G′amez‐Meza, 2010) where high contents of gallic acid and flavonoids were found in the grape skin extracts with high antioxidant activities.

## 4 DISCUSSION

In this study, the obtained results revealed that the wasted parts, particularly the peels of lemon, orange, and grapefruit, contain appreciable amounts of phenolic compound and vitamin C as well as high antioxidant activity rate. According to Ghafoor, Al‐Juhaimi, and Choi (2011), phenolic compounds from natural resources are recommendable as food additives in food processing more than the artificial antioxidants butylated hydroxyanisole and butylated hydroxytoluene. Moreover, the addition of phenolic in food is also reported for its nutritional and health benefits (Dalar, Türker, Zabaras, & Konczak, 2014).

From Table 1, it is clearly observed that high correlation between the metabolite in the peels and whole fruit. It was found positively higher in the peel than in the inner part waste of all citrus fruit used in this study. On the other hand, among the metabolites, total phenolic content had a higher correlation to the antioxidant activity, particularly in the peels, than the other one (Table 2). A positive correlation between antioxidant activity and total phenolic content indicated that phenolic could be one of the main contributors to the antioxidant capacities of these fruit residues. The values of phenolic compounds in peels were higher than those in pulps, indicating that they could be inexpensive and readily available resources of bioactive compounds (such as natural antioxidant) for use in the food and pharmaceutical industries. However, Arena, Fallico, and Maccarone (2001) stated that phenolic compounds in citrus fruits contributed less than vitamin C in establishing the antioxidant power. Most of the studies revealed that the activity of antioxidants is governed to a larger extent by phenolic compounds than ascorbic acid particularly in that of plant sources Manganaris, Goulas, Vicente, and Terry (2013) and Silva, O'Callagahan, O'Brien, and Netto (2013). These distinctive inferences could be occurring due to the difference according to the cultivar types besides various variable factors include maturity of fruit and the analytical methods used in various studies for estimation antioxidant power.

**Table 1:** Correlation of total phenolic, total flavonoids, vitamin C, and antioxidant activity between wasted parts and whole fruits of citrus

Wasted parts	Coefficient of determination (*R* ^2^)			
	Total phenolic	Total flavonoids	Vitamin C	Antioxidant activity
Peels	.9416	.9674	.9865	.6547
Pulp and seeds	.4563	.0265	.9851	.0311

**Table 2:** Correlation between antioxidant activity (DPPH) and level of total phenolic, total flavonoids, and vitamin C in wasted parts of citrus fruits

Metabolite	Coefficient of determination (*R* ^2^)	
	Peels	Pulp and seeds
Total phenolic	.9303	.1734
Total flavonoids	.1001	.0694
Vitamin C	.2021	.2796

DPPH, diphenyl‐2‐picrylhydrazyl.

In general, our findings concluded that the wasted parts of Sudanese citrus fruits, particularly the peels, considered as good sources of phenolic compounds with excellent radical scavenging properties. Nevertheless, the exploitation of citrus by‐products is yet to be a mature industry due to several challenges. Therefore, further researches and studies are needed before conducting these natural resources from waste to the food industries.

## 5 CONCLUSION

In this study, phenolic composition, vitamin C content, and antioxidant activity of the wasted part (peel and pulp with seeds) of different citrus fruit grapefruit, orange, and lemon have been examined. The obtained results explored that total phenolic content, flavonoids, vitamin C, and antioxidant activity of lemon, orange, and grapefruit peels were higher than in those of their pulp and seeds. Among studied citrus fruit, the capacity of the antioxidant and its activity were found much higher in grapefruit followed by lime and orange.

Therefore, prudent use of by‐products from the wasted part of citrus fruits can also be helpful for maximum utilization of natural foods and at the same time assist in environmental protection.
